# Follicle-stimulating hormone receptor gene polymorphism in chronic anovulatory women, with or without polycystic ovary syndrome: a cross-sectional study

**DOI:** 10.1186/1477-7827-12-86

**Published:** 2014-09-02

**Authors:** Wanakan Singhasena, Tawiwan Pantasri, Waraporn Piromlertamorn, Sudarat Samchimchom, Teraporn Vutyavanich

**Affiliations:** Division of Reproductive Medicine, Department of Obstetrics and Gynecology, Faculty of Medicine, Chiang Mai University, Chiang Mai, 50200 Thailand

**Keywords:** FSH receptor, Polymorphisms, PCOS, Anovulation

## Abstract

**Background:**

Polymorphisms at codons 307 and 680 are the most commonly encountered allelic variants of the follicle-stimulating hormone receptor (FSHR) gene. Studies in Caucasians suggest that certain FSHR variants are more common in women with polycystic ovary syndrome (PCOS) than normal women. The objective of this study was to determine the distribution of FSHR gene polymorphisms at codons 307 and 680 in Thai women with chronic anovulation, without (121 women) and with PCOS (133 women), using 132 known fertile women as controls.

**Methods:**

DNA samples from peripheral blood lymphocytes were extracted and analyzed by polymerase chain reaction-restriction fragment length polymorphism.

**Results:**

The prevalence of Threonine307Threonine (TT), Threonine307Alanine (TA), and Alanine307Alanine (AA) genotypes at codon 307 was 53.0% (95% CI = 44.2-61.7%), 42.4% (95% CI = 34–51.3%), and 4.5% (95% CI = 1.9-10.1%) in controls; 52.6% (95% CI = 43.8-61.3%), 39.8% (95% CI = 31.6-48.7%), and 7.5% (95% CI = 3.9-13.7%) in PCOS women; and 50.4% (95% CI = 42.8-61.2%), 45.4% (95% CI = 34.9-53.1%), and 4.5% (95% CI = 1.5-9.6%) in anovulatory women without PCOS, respectively. The prevalence of Asparagine680Asparagine (NN), Asparagine680Serine (NS), and Serine680Serine (SS) genotypes at codon 680 was 54.5% (95% CI = 45.7-63.2%), 40.9% (95% CI = 32.5-49.8%), and 4.5% (95% CI = 1.9-10.1%) in controls; 51.9% (95% CI = 43.1-60.6%), 44.4% (95% CI = 35.8-53.2%), and 3.8% (95% CI = 1.4-9.0%) in PCOS women; and 47.9% (95% CI = 40.4-58.8%), 47.1% (95% CI = 36.5-54.7%), and 5.0% (95% CI = 2–10.9%) in anovulatory women without PCOS, respectively. The prevalence of FSHR gene polymorphisms at both codons were not statistically different among the three groups.

**Conclusions:**

In Thai women, there was no association between the FSHR gene polymorphism at codons 307 and 680 and chronic anovulation.

## Background

Chronic anovulation is a common disorder in women of reproductive age. Some women with chronic anovulation also have high androgen levels or polycystic ovary appearance on ultrasound examination, and are diagnosed as having polycystic ovary syndrome (PCOS) by the 2003 Rotterdam criteria [1]. The pathogenesis of chronic anovulation, with or without PCOS, is poorly understood. A high incidence of similar phenotypes in family members of PCOS patients suggests that genetics may play a role [2]. Though the polymorphisms in genes encoding sex hormones and their receptors have been investigated, results are still conflicting [3].

Follicle stimulating hormone (FSH) plays an important role in follicular growth and ovarian steroidogenesis. Mutations or polymorphisms in the FSH receptor (FSHR) gene can affect reproductive ability [4]. The FSHR gene contains two important single nucleotide polymorphisms (SNPs) in exon 10, which are in linkage disequilibrium. One polymorphism is located at codon 307 in the extracellular domain of the receptor, where alanine is replaced by threonine (A307T; rs6165). The other polymorphism is in the intracellular domain at codon 680, where asparagine is replaced by serine (N680S; rs6166) [5, 6]. To date, few genetic studies have examined the association between FSHR polymorphisms and PCOS [7–11]. Sudo *et al*. [9] reported a significant increase in the A307T and N680S genotypes in a large cohort of Japanese women with PCOS compared to ovulatory women. Some studies reported that the S680S genotype in PCOS women was associated with higher baseline gonadotropic hormone and testosterone levels [12], as well as clomiphene citrate resistance [13]. Since PCOS prevalence and clinical manifestations, as well as frequency of the FSHR polymorphisms, can differ between ethnic and racial groups [14–20], more studies should be done to confirm this finding.

The objective of this study was to compare the prevalence of the FSHR gene polymorphisms at codons 307 and 680 in Thai women with chronic anovulation, with PCOS (group I) and without PCOS (group II). We used pregnant women, who had regular cycles and achieved spontaneous pregnancies within 12 months of unprotected intercourse as controls (group III). The results of the study could suggest whether the FSHR polymorphisms at these two codons played a role in chronic anovulation, and whether we could use them to differentiate chronic anovulatory women with and without PCOS.

## Methods

### Study and control population

The study was approved by the Ethics Committee of the Faculty of Medicine, Chiang Mai University (No. 012/2011, dated January 7, 2011). Written informed consent, previously approved by the Institutional Review Board, was read and signed by all participants. The study population consisted of women with chronic anovulation (*n* = 121) and PCOS (*n* = 133), who attended either the gynecologic outpatient clinic or gynecologic endocrine clinic, Maharaj Nakorn Chiang Mai University Hospital from January 2012 to August 2013. The controls were 132 pregnant women, who first visited the antenatal care clinic in their first trimesters during the same time period as women with chronic anovulation.

In the anovulation group, women were included if they were Thai, aged 20 to 40 years, had both ovaries present on ultrasound scan, had irregular menstrual cycles, and oligomenorrhea (>35 day intervals between periods), or amenorrhea (absence of vaginal bleeding for at least 6 months or 3 cycles of normal periods), which was persistent for at least 1 year. They were excluded if they: 1) used oral contraceptive pills, hormonal intrauterine devices (IUDs) or other hormonal therapy within 3 months prior to the study; 2) had other endocrine disorders, such as diabetes mellitus, thyroid diseases, premature ovarian failure, hyperprolactinemia or other known causes of androgen excess disorders; 3) had language or other communication barriers; 4) had previous history of ovarian surgery; 5) had previous exposure to cytotoxic drugs or pelvic radiation therapy; and 6) did not consent to participate in the study. Anovulatory women were classified as having PCOS if they fulfilled the Rotterdam criteria (1); otherwise they were classified as having chronic anovulation without PCOS.

The controls consisted of 132 pregnant women who fulfilled the following eligibility criteria: Thai women, aged 20 to 40 years, first attendance at the antenatal care clinic, had regular menstrual cycles with intervals of 21 to 35 days, and spontaneous pregnancy within 12 months of attempt. They were excluded if they: 1) had a history of infertility or any infertility treatment in the past; 2) had language or other communication barriers; 3) had a gestational age of ≥10 weeks; and 4) did not consent to participate in the study.

### Evaluations and hormone assays

Anovulatory subjects underwent routine examinations, which included a detailed history, physical and pelvic examination, transvaginal ultrasound, and other laboratory studies as deemed necessary by the attending gynecologists. In addition, blood samples were obtained for hormonal studies and DNA analysis. Plasma concentrations of follicle stimulating hormone (FSH), luteinizing hormone (LH), estradiol (E_2_), prolactin (PRL), testosterone (T), and sex hormone binding globulin (SHBG) were measured with electrochemiluminescence immunoassays (Elecsys®, Roche Diagnostics, Indianapolis, USA). Plasma concentrations of androstenedione (ADD), 17-hydroxyprogesterone (17-OHP), dehydroepiandrosterone sulfate (DHEAS) were analyzed using Enzyme-linked Immunosorbent Assay (ELISA, NovaTec Immundiagnostica, GmbH, Germany). Intra- and inter-assay coefficients of variation of all hormonal assays were <4.5% and <6.5%, respectively. Any patient having 17-OHP levels higher than 4.0 ng/mL received ACTH stimulation test to exclude late-onset congenital adrenal hyperplasia.

Subjects in the control group had routine physical and obstetrical examinations, and blood tests as usual for those attending the antenatal clinic for the first time. In addition, three milliliters of blood were obtained in tubes containing ethylenediaminetetraacetic acid for DNA analysis.

### Genotyping

DNA was extracted from peripheral blood leukocytes, using QIAamp DNA kit (QIAGEN, Hilden, Germany), according to the manufacturer’s instructions, and kept frozen at -20°C until use. Genotyping was performed without knowledge of the clinical status of the subjects.

**Figure 1:**
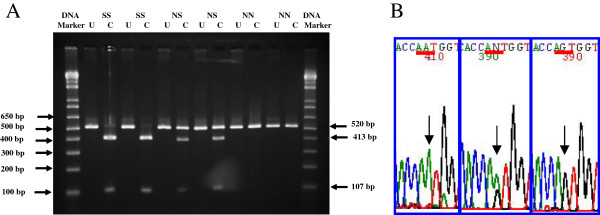
**Agarose gel electrophoresis. (A)** Restriction fragment length polymorphism (RFLP) analysis of the Asn680Ser FSH receptor variant. Agarose gel (2.0%) electrophoresis with ethidium bromide staining following *BsrI* digestion of the PCR products is shown. A 520-bp band for *NN*, two bands of 520 and 413-bp for *NS*, and a 413-bp band for *SS*, are shown. **(B)** DNA sequencing of each variant PCR products. Positions of the nucleotide substitutions are indicated by arrows.

A part of exon 10, from nucleotides 1624 to 2143 of the FSHR gene, was amplified by Polymerase Chain Reaction (PCR), using forward and reverse primers as follows: (primer-1) 5′-TTTGTGGTCATCTGTGGCTGC-3′ and (primer-2) 5′-CAA AGGCAAGGACTGAATTATCATT-3′. The PCR reactions were performed in an Eppendorf Personal Cycler Thermocycler (Eppendorf, Germany) with 5 uL Taq polymerase PCR Master Mix (Invitrogen, USA), 20 pmols of each primer and 100 ng of DNA in a final volume of 10 μL. The cycling conditions were as follows: initial denaturation at 94°C for 5 minutes, 30 cycles consisting of denaturation at 95°C for 30 seconds, annealing at 60°C for 30 seconds, extension and final elongation at 72°C for 7 minutes. The PCR products were digested with *BsrI* (Promega, USA), an endonuclease that recognized the A to G transition sites. The resulting products were run on 2% agarose gel electrophoresis, stained with ethidium bromide and photographed. Three possible patterns were expected (Figure 1): 1) a single band of undigested products of 520 base pairs in size, indicating homozygous Asn680Asn (NN); 2) a single band of digested products of 413 base pairs, indicating homozygous Ser680Ser (SS), and two bands of 520 and 413 base pairs, representing heterozygous state for Asn680Ser (NS). Ten percent of the samples were randomly selected for DNA sequencing to confirm the presence of polymorphisms shown by PCR-Restriction Fragment Length Polymorphism (PCR-RFLP) analysis.

**Figure 2:**
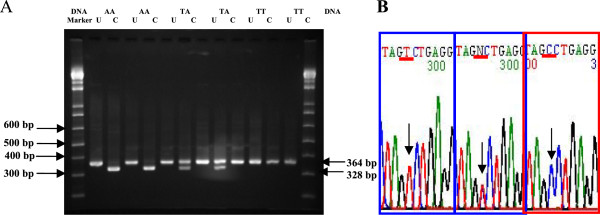
**Agarose gel electrophoresis. (A)** Restriction fragment length polymorphism (RFLP) analysis of the Thr307Ala FSH receptor variant. Agarose gel (2.0%) electrophoresis with ethidium bromide staining, following *Bsu36I* digestion of the PCR products. A 364-bp band for TT; two bands, 364- and 328-bp, for TA; and a 328-bp band for AA, are shown. **(B)** DNA sequencing of each variant PCR products. Positions of the nucleotide substitution are indicated by arrows.

Detection of the Thr307Ala variant was performed by a nested PCR-RFLP method. In this case, a second round of PCR, using a second set of primers that bound to a secondary target within the first run products, was necessary to reduce non-specific products generated by the first run of PCR. The region of exon 10, containing nucleotides 931 to 1587 of the FSHR gene, was first amplified using forward and reverse primers as follows: (primer-3) 5′-TCTGAGCTTCATCCAATTTGCA-3′; and (primer-4) 5′-GGGAAAGAGGGCAGCTGCAA-3′. The PCR reactions were performed in an Eppendorf Personal Cycler Thermocycler with 5 uL Taq polymerase PCR Master Mix, 20 pmols of each primer and 100 ng of DNA in a final volume of 10 μL. The cycling conditions were as follows: initial denaturation at 94°C for 5 minutes, followed by 10 cycles of denaturation at 94°C for 1 minute, annealing at 62°C for 1 minute, extension at 72°C for 1 minute, and a final elongation at 72°C for 7 minutes. The PCR products (657 base pairs of DNA fragments) after the first round (2 μL) were further amplified by a second set of primers as follows: (primer-5) 5′-CAAATCTATTTTAAGGCAAGAAGTTGATTATATGCC TCAG-3′ and (primer-6) 5′-GTAGATTCCAATGCAGAGATCA-3′. The PCR cycle was carried out for 1 minute at 94°C for initial denaturation, followed by 25 cycles of denaturation at 94°C for 30 seconds, annealing at 60°C for 30 seconds, extension at 72°C for 30 seconds, and a final elongation step at 72°C for 7 minutes. The final PCR products were digested with *Bsu36I* restriction enzyme, which recognized the transition sites of T to C. Restriction endonuclease digestion products were visualized on 2% agarose gel, stained with ethidium bromide, and photographed. Three different patterns were possible (Figure 2): 1) a single band of undigested products of 364 base pairs in size, indicating a homozygous state for Thr307Thr (TT); 2) a single band of digested products of 324 base pairs in size, indicating a homozygous state for Ala307Ala (AA); and 3) two bands of 364 and 324 base pairs, indicating a heterozygous state for Thr307Ala (TA). Ten percent of samples were randomly selected for DNA sequencing to confirm the results obtained from PCR-RFLP analysis.

### Statistical analysis

Descriptive statistics (mean ± SD) were used to summarize baseline characteristics and hormone levels of patients in the controls and the two study groups. The prevalence of FSHR polymorphism, and its 95% confidence interval, was calculated in each group, and compared among groups using rxc Chi-Square tests. All statistical analyses were performed using SPSS version 15 for windows (IBM company, IL, USA). A value of *P* < 0.05 was considered to indicate statistical significance.

In a previous study in Japanese women [9], the prevalence of NS allele in PCOS women was significantly higher than that in the ovulating group (66.7% versus 43.5%, P < 0.05). Given this difference, and a type I error of 0.05 (two-tailed) and a power of 80%, we calculated that a sample size of 71 women would be required per group. To ensure a sufficient sample size, we planned to recruit at least 100 subjects with PCOS and 100 controls.

## Results

A total of 950 women with oligomenorrhea or amenorrhea were screened, and 308 women met the eligibility criteria for the study. Of these 121/151 and 133/157 women with chronic anovulation and PCOS, respectively, consented to participate in the study. There was 100% agreement between the results obtained by PCR-RFLP and DNA sequencing in the 10% of random samples that were sent out for confirmation.

The allelic frequencies for the polymorphisms at codons 307 and 680 in controls, women with PCOS, and women with chronic anovulation without PCOS are shown in Table 1. The prevalence of polymorphism obtained at the codons Thr307Ala and Asn680Ser were similar across all groups. There was no statistical difference in the frequency distribution of these genotypes at codon 307 (*P* = 0.756) or 680 (*P* = 0.932) among fertile women and those with anovulation, with or without PCOS.

**Table 1:** **FSHR gene polymorphism at positions 307 and 680 in PCOS**, **chronic anovulatory**, **and control women**

	Position 307			Position 680		
	Thr/Thr	Thr/Ala	Ala/Ala	Asn/Asn	Asn/Ser	Ser/Ser
**PCOS**	52.6%	39.8%	7.5%	51.9%	44.4%	3.8%
(95% CI) (n = 133 women)	(43.8%-61.3%)	(31.6%-48.7%)	(3.9%-13.7%)	(43.1%-60.6%)	(35.8%-53.2%)	(1.4%-9.0%)
**Chronic anovulation**	50.4%	45.4%	4.5%	47.9%	47.1%	5.0%
(95% CI) (n = 121 women)	(42.8%-61.2%)	(34.9%-53.1%)	(1.5% -9.6%)	(40.4%-58.8%)	(36.5%-54.7%)	(2.0%-10.9%)
**Controls**	53.0%	42.4%	4.5%	54.5%	40.9%	4.5%
(95% CI) (n = 132 women)	(44.2%-61.7%)	(34.0%-51.3%)	(1.9%-10.1%)	(45.7%-63.2%)	(32.5%-49.8%)	(1.9%-10.1%)

There was no statistically significant difference in age, serum FSH, estradiol, and PRL levels between anovulatory women with or without PCOS (Table 2). However, BMI, serum LH, ADD, DHEAS, 17-OHP, testosterone, and free androgen index (FAI) were significantly higher in the PCOS group than the anovulatory group without PCOS, and serum SHBG level was significantly lower.

**Table 2:** **Demographics and endocrine profiles of anovulatory women**, **with or without PCOS**

	Anovulatory women (n = 121)	PCOS (n = 133)	P*
Age (years)	27.4 ± 5.6	26.6 ± 5.3	0.2092
Body mass index (kg/m^2^)	21.2 ± 5.6	24.3 ± 6.1	<0.0001
*Serum hormones*			
Follicle stimulating hormone (mIU/mL)	5.5 ± 3.2	6.1 ± 2.4	0.143
Luteininzing hormone (mIU/mL)	9.7 ± 7.7	12.2 ± 7.3	0.0100
Estradiol (pg/mL)	106.1 ± 140.1	86.8 ± 130.1	0.2546
Prolactin (ng/mL)	16.8 ± 11.5	15.3 ± 8.8	0.2398
Androstendione (ng/mL)	1.7 ± 1.6	2.5 ± 1.3	<0.0001
Dehydroepiandrosterone sulfate (ng/mL)	1.4 ± 1.0	2.4 ± 1.3	<0.0001
17-hydroxyprogesterone (ng/mL)	1.1 ± 1.6	1.6 ± 1.5	0.0052
Testosterone (ng/mL)	0.3 ± 0.1	0.6 ± 0.3	<0.0001
Sex hormone binding globulin (nmol/L)	69.8 ± 47.4	40.8 ± 29.7	<0.0001
Free androgen index (FAI)	0.6 ± 0.6	2.6 ± 2.5	<0.0001

Values = mean ± standard deviation.

*t-tests.

*PCOS* = polycystic ovary syndrome; *mIU* = milli International Unit; *mL* = milliliter; *pg* = picogram; *ng* = nanogram; nmol = nanomol; *L* = liter.

Subgroup analysis was performed by pooling anovulatory subjects with and without PCOS together, and classified them by their FSHR genotypes (Table 3). When polymorphism at codon 307 was considered, there were statistical significant differences in the levels of FSH (*P* <0.001) and LH (*P* = 0.003), but not in their ages, estrogen level or androgen profiles. In case of the polymorphism at the codon 680, only the level of LH was statistically different (*P* = 0.041).

**Table 3:** **Anovulatory subjects** (**with and without PCOS**) **classified by FSHR genotypes at codon position 307 or 680**

Variable	Polymorphism at codon 307				Polymorphism at codon 680			
	TT (n = 131)	TA (n = 108)	AA (n = 15)	P*	NN (n = 127)	NS (n = 116)	SS (n = 11)	P*
Age (range) in year	27.0 (20–40)	26.5 (20–40)	28.4 (20–38)	0.419	27.1 (20–40)	26.6 (20–38)	27.9 (21–40)	0.639
***Serum hormone***								
FSH (mIU/mL)	5.4 ± 2.6	6.2 ± 2.9	9.2 ± 9.7	0.000	5.5 ± 2.5	6.5 ± 4.5	5.8 ± 2.7	0.106
LH (mIU/mL)	9.4 ± 6.2	12.6 ± 8.6	12.7 ± 6.6	0.003	9.9 ± 6.8	12.3 ± 8.2	9.2 ± 7.0	0.041
Estradiol (pg/mL)	92.7 ± 138.3	93.9 ± 106.6	95.3 ± 121.1	0.995	91.8 ± 137.7	97.6 ± 113.2	61.5 ± 46.7	0.646
Prolactin (ng/mL)	16.5 ± 10.8	16.4 ± 11.5	13.9 ± 7.4	0.694	16.5 ± 10.8	15.9 ± 11.1	17.8 ± 9.8	0.822
ADD (ng/mL)	2.1 ± 1.2	2.1 ± 1.3	2.4 ± 1.1	0.653	2.1 ± 1.2	2.1 ± 1.2	2.0 ± 1.5	0.938
DHEAS (ng/mL)	2.1 ± 1.3	2.2 ± 1.6	2.9 ± 2.3	0.124	2.1 ± 1.4	2.3 ± 1.7	1.6 ± 0.9	0.169
17-OHP (ng/mL)	1.3 ± 1.4	1.2 ± 0.9	1.5 ± 0.9	0.680	1.3 ± 1.4	1.3 ± 0.9	0.9 ± 0.7	0.463
Testosterone (ng/mL)	0.5 ± 0.3	0.5 ± 0.4	0.5 ± 0.4	0.233	0.5 ± 0.3	0.6 ± 0.4	0.3 ± 0.2	0.067
SHBG (nmol/L)	52.5 ± 43.3	56.4 ± 41.5	30.7 ± 15.0	0.474	52.7 ± 42.8	55.5 ± 43.3	46.5 ± 25.6	0.826

*oneway ANOVA test.

## Discussion

Kuijper *et al*. [20] reported the distribution of single nucleotide polymorphisms (SNPs) at codon 680 of the FSHR gene in a large population of infertile patients (1,771 women) from various ethnic backgrounds. Of these, only 3.8% or 67 women were Asians and only 11% of them had cycle disorders. They found a significantly lower prevalence of Ser680Ser FSHR variant (10.4%), and a significantly higher prevalence of Asn680Asn variant (50.7%) in Asians, compared with Caucasians and Mediterranean. These findings were compatible with our study in Thai women. Similar to their study, we found no difference in FSH concentration among the various FSHR polymorphisms at this position. Unlike other previous studies, where basal FSH and LH levels were measured on cycle day 2 or 3, we measured random levels of FSH and LH during the anovulatory period. Interestingly, we found that serum FSH and LH levels were significantly higher in anovulatory subjects who carried the Thr307Ala or Ala307Ala genotypes. This finding needs to be confirmed by further studies in a larger population and from other ethnic groups. We hypothesized that SNP at this codon, which was involved in FSH binding, could result in a less active receptor. As a consequence, a higher amount of FSH was required to generate the same downstream effect as a woman with Thr307Thr genotype (wild type). The increase in FSH level was probably driven by an increase in hypothalamic gonadotropin releasing hormone activity, which concomitantly resulted in a higher LH secretion. The slight increase in gonadotropin levels was probably not clinically significant under normal physiological conditions, as the distribution of FSHR polymorphisms was not different among fertile controls with regular cycles and those with chronic anovulation. However, polymorphisms at these two codons could interact with polymorphisms of other genes that were involved in steroid productions and folliculogenesis, and resulted in anovulation or an abnormal response to ovarian stimulation during an assisted conception treatment.

In a study of 522 Japanese women, Sudo *et al*. [9] found significantly higher prevalence of Asn680Ser genotypes in Japanese PCOS women than in those who had regular ovulation. However, their study contained only 18 PCOS women. Our study, which consisted of 133 Thai PCOS women, did not confirm their findings. Conway *et al*. [7] also reported no difference in the prevalence of Thr307/Ser680 allelic variants of FSHR gene among 93 British PCOS women and 51 controls.

In Dutch women, Laven *et al*. [21] reported a significantly lower prevalence of Thr307Thr genotype and significantly higher prevalence of the Ser680Ser polymorphism in 148 anovulatory infertile women (61 of whom had PCOS) than controls. Our study, with 254 anovulatory women, did not confirm their findings. This discrepancy could be due to the difference in ethnic group, clinical presentation, or simply because of random errors as they had fewer cases and only 30 controls. Indeed, another recent study involving 580 anovulatory Dutch women, of whom 518 were diagnosed with PCOS and 2,996 unselected controls from the general population, could not find any difference in the distribution of FSHR genotypes at position 680 [12]. On the other hand, a recent meta-analysis in women with PCOS showed that the FSHR Ala307/Ser680 genotype was associated with an increased risk, while homozygous for Asn680Asn showed a significant reduction in PCOS risk [22]. However, in this meta-analysis only 3 out of the 8 included studies, with 18, 50 and 93 PCOS women, showed a significant difference in genotype profiles. If our study was included in this meta-analysis, results would shift direction toward no association.

The difference in androgen profiles between anovulatory women with and without PCOS in our study was most likely due to classification bias, as hyperandrogenemia is one of the diagnostic criteria for PCOS. In addition, the higher BMI and LH were common features, although not unique, in PCOS cases.

Although our study did not show any difference in the distribution of FSHR genotypes at positions 307 and 680, it was possible that a very small difference could exist. Our calculation showed we had a statistical power of 20.6% (given a Type I error of 5%, two-tailed) to show a significant difference between the prevalence of 4.5% versus 7.5% in Ala/Ala genotype at position 307 in the chronic anovulation and the PCOS group, respectively. A larger sample size of 1,200 or more women with chronic anovulation, with or without PCOS, will need to be recruited to ascertain that there was no subtle genotype difference between groups at this position.

The strength of our study was that we used hormonal profiles and ultrasound, in addition to more subjective clinical signs and symptoms, to classify chronic anovulatory patients into those with and without PCOS. We identified SNPs using PCR-RFLP, which is known to have a very high diagnostic accuracy [23, 24] , and we randomly confirmed our PCR-RFLP results by sequencing.

## Conclusions

This was the first study to report FSHR polymorphisms at position 307 and 680 in Thai women with chronic anovulation, with and without PCOS, compared to normal fertile controls with regular ovulation. In contrast to a previous study in Japanese women, we found no association between FSHR gene polymorphisms at positions 307 and 680 and chronic anovulation, or PCOS.

## Abbreviations

A307A or AA: Alanine and alanine at position 307
A307T or AT: Alanine and threonine at position 307
ACTH: Adrenocorticotrophic hormone
680Asn/Asn or NN: Asparagine and asparagine at position 680
BMI: Body mass index
Bp: Base pair
DHEAS: Dehydroepiandrosterone sulfate
DNA: Deoxyribonucleic acid
E_2_: Estradiol
EDTA: Ethylenediaminetetraacetic acid
FAI: Free androgen index
FSH: Follicle stimulating hormone
Elecys: Electrochemiluninescence
ELISA: Enzyme-linked immunosorbent assay
FSHR: Follicle stimulating hormone receptor
IUDs: Intrauterine contraceptive devices
LH: Luteinizing hormone
N680S or NS: Asparagine680Serine
17-OHP: 17-hydroxyprogesterone
PCOS: Polycystic ovary syndrome
PCR: Polymerase chain reaction
PRL: Prolactin
RFLP: Restriction fragment length polymorphism
SD: Standard deviation
680Ser/Ser or SS: Serine and serine at position 680
SHBG: Sex hormone binding globulin
SNPs: Single nucleotide polymorphisms
SPSS: Statistical package for social science
T: Testosterone
Thr307Thr or TT: Threonine and threonine at position 307.
